# IL-17A drives a fibroblast-neutrophil-NET axis to exacerbate immunopathology in the lung with diffuse alveolar damage

**DOI:** 10.3389/fimmu.2025.1574246

**Published:** 2025-06-11

**Authors:** Duo Su, Lu Li, Hao Xie, Lingli Ai, Yuqing Wang, Bo Yang, Dongsheng Zhou, Lingfei Hu, Huiying Yang

**Affiliations:** ^1^ State Key Laboratory of Pathogen and Biosecurity, Academy of Military Medical Sciences, Beijing, China; ^2^ Reproductive Genetics Center, Bethune International Peace Hospital, Shijiazhuang, China

**Keywords:** diffuse alveolar damage, neutrophil extracellular trap, IL-17A, fibroblast, scRNA-seq

## Abstract

Diffuse alveolar damage (DAD), a lethal manifestation of acute lung injury, remains a critical public health concern due to the absence of targeted therapies. However, the underlying cellular and molecular mechanisms responsible for immunopathology during DAD progression are largely undefined. Here, by integrating single cell RNA sequencing, functional assays, and genetic/pharmacological interventions in a mouse model of ricin-induced DAD, we revealed a significant accumulation of neutrophil with an activated phenotype that plays a critical role in immunopathology. We observed the formation of neutrophil extracellular traps (NETs) during DAD, which further intensified inflammation and tissue injury. IL-17A signaling activity was upregulated in DAD-affected lungs, while IL-17A deficiency or functional blockade significantly attenuated neutrophil recruitment, NET generation, and tissue damage. Mechanically, IL-17A stimulates lung resident fibroblasts to produce the neutrophil chemoattractant CXCL1. Notably, type 3 innate lymphoid cells (ILC3) emerged as the dominant source of IL-17A, highlighting a triad of interactions among ILC3, fibroblast, and neutrophil in DAD pathogenesis. This finding delineates a pathogenic IL-17A-neutrophil-NET axis that amplifies lung immunopathology after ricin-induced DAD, a deeper understanding of these relationships may pave the way for mitigate DAD immunopathology and other lung inflammatory disorders.

## Graphical Abstract

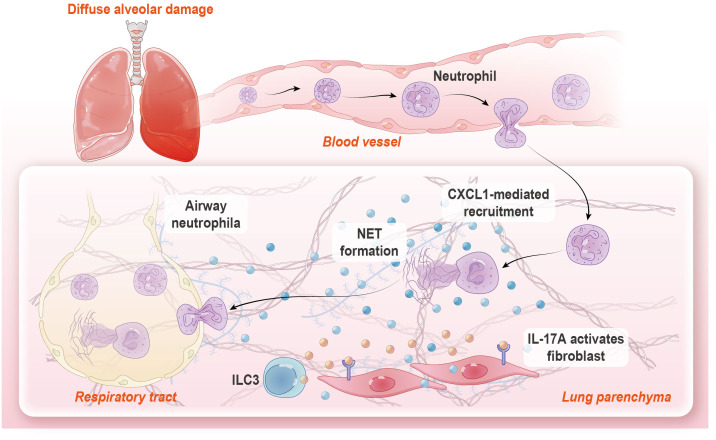

## Highlights

- IL-17A orchestrates neutrophilic response in DAD immunopathology.
- NET formation exacerbates DAD pathology.
- Fibroblast-derived CXCL1 fuel neutrophil chemotaxis.
- ILC3 emerges as a key IL-17A source in DAD progression.

## Introduction

Diffuse alveolar damage (DAD), a histopathological hallmark of severe acute lung injury and interstitial pneumonia, arises from complex pathogenic mechanisms primarily driven by dysregulated inflammatory cascade activation. DAD are clinically characterized by acute respiratory failure with a high mortality rate of 50% (1). The high mortality rate is largely due to limited effective treatment options, while current therapeutic approaches remain predominantly supportive, focusing on globe anti-inflammatory interventions. Since no effective therapeutics have been developed for this severe respiratory disorder, understanding the molecular and cellular mechanisms of DAD progression is key to the development of novel drugs targeting the prevention of hyperinflammation.

Massive infiltration of immune cells, especially neutrophil, play a significant role in aggravating the lung immunopathology during lung inflammatory disease. Neutrophil could exhibit cytotoxic effect though neutrophil extracellular trap (NET) formation, cytokine production, and impaired degranulation, which aggravate tissue damage (2–5). NETs are web-like structures composed of DNA along with antimicrobial components, such as histone (Cit-H3) and myeloperoxidase (MPO). These structures are generated via PAD4-dependent histone citrullination that contribute to chromatin decondensation by MPO. NETs unequivocally trap and kill various invading pathogens, yet excessive NET formation is implicated in exacerbating tissue damage, especially in the sterile inflammation (6). However, the mechanisms that regulated neutrophil recruitment and NET formation are still incompletely understood. Recent evidence suggests that lung stromal cells, such as fibroblasts, are involved in shaping the immune environment (7–9). Following an injury, silenced fibroblasts are activated and produce a range of immunoregulatory factors, including chemokines and cytokines, which orchestrate neutrophil recruitment and activation (10). Despite this knowledge, the specific cues that prompt fibroblasts to engage in pathological activities remain unclear.

The IL-17 family consists of multiple pro-inflammatory proteins (11). Among these, IL-17A is often found at elevated levels in various infectious disorders, autoimmune diseases, and several types of cancer (12–14). It plays a crucial role in the immune response by enhancing the recruitment of immune cells from the bloodstream while also directly boosting the function of tissue resident cells. This dual action contributes to protective immunity against pathogen challenge, but it can also lead to immunopathological damage (15, 16). Notably, IL-17A can be produced not only by innate lymphocytes such as NK cells, NKT cells, γδ T cells, and type 3 innate lymphoid cells (ILC3s), but also adaptive lymphocytes like CD8^+^ and CD4^+^ T cells (17). However, whether IL-17A participate the immunopathologic process after DAD has yet to be fully elucidated.

Previous research in our lab demonstrated that fibroblast and neutrophil constituted an anatomically localized inflamed niche in peribranchial regions after ricin-induced DAD in mice (18). In this study, through single-cell RNA sequencing (scRNA-seq), functional assays, and genetic/pharmacological interventions, we uncovered a self-amplifying IL-17A-fibroblast-neutrophil axis that exacerbates tissue damage through NET in this condition. Mechanistically, IL-17A stimulated lung resident fibroblast to produce CXCL1, a potent neutrophil chemoattractant to sustain neutrophilic inflammation and NET formation. Notably, type 3 innate lymphoid cells (ILC3s) emerged as the dominant source of IL-17A in DAD-affected lungs (**Graphical Abstract**). These findings extend our prior characterization of inflammatory microenvironments in DAD immunopathology, which could offer potential targeted strategies for DAD treatment.

## Materials and methods

### Animal model

C57BL/6J mice were purchased from HFK Bioscience Co Ltd., Beijing, China. *Il17a* gene knock-out mice (*Il17a* ^KO^, C57BL/6j background) were obtained from GemPharmatech Technology Co. Ltd., Nanjing, China. All mice were housed and maintained in specific pathogen-free conditions with a 12:12 light/dark schedule, controlled ambient temperature, and were given free access to food and water. All animal experiment reports in this study comply with the ARRIVE guidelines. Ethical approval for all experiments was obtained from the Institutional Animal Care and Use Committee (Approval number: IACUC-DWZX-2024-003).

Female mice, 6–8 weeks old, were used to generate the mouse model of DAD, as previously described (19–21). Briefly, Mice were subjected to pentobarbital sodium intraperitoneally anesthesia and placed in a supine position. Aerosolized ricin (approximately 0.01 mg/kg) in 50 µL phosphate-buffered saline (PBS) was delivered to the mice intratracheally using a MicroSprayer aerosolizer (Huironghe Company). Control groups were treated with an identical volume of PBS. All procedure were conducted in biosecurity level 3 laboratories. For neutrophil depletion, mice were injected intraperitoneally with anti-Ly6G antibody (1A8, BioXcell, 200 μg/mouse) 24 h prior to and 24 h after challenge. Corresponding isotype antibody (IgG2b, BioXcell) was used as a control. For NET inhibition, mice were injected intraperitoneally with DNase I (Sigma-Aldrichm) and Cl-Amidine (MedChemExpress) immediately following ricin exposure with another dose administration at 24 h after injury. Mice, receiving equivalent volumes of PBS or DMSO and PBS mixture (1:1), were used as vehicle control, respectively. The sample size for each group in every experiment is specified in the figure legends.

### Sample collection

Mice were sacrificed by excessive anesthesia i.p. at the indicated time-points after ricin-induced DAD. BALF was acquired using 800 μL of cold PBS through the murine trachea. BALF was centrifuged (500 × g, 5 min), the separated BALF supernatant was used to measure total protein, cytokine, chemokine, and Cit-H3 levels. The BALF pellets were used for flow cytometry analysis. Total protein concentrations in BALFs were assessed by the Pierce BCA Protein Assay Kit (Thermo Scientific). The cytokine and chemokine including IL-17A and CXCL1, in BALFs were quantified using ELISA kits (Solarbio). For detecting NETs, citrullinated histone3 (Cit-H3) in BALF were detected using ELISA kits (ELK Biotechnology). The lungs were extracted for histopathological evaluation, immunofluorescence staining, and flow cytometry analysis.

### Cell culture and treatment

MLg cells (murine lung fibroblast line, ATCC CCL-206TM) were purchased from the Tong Pai Technology Biological company (Shanghai, China). Cells were maintained in Dulbecco’s modified Eagle’s medium (DMEM, Gibco, California, US) supplemented with 10% fetal bovine serum (Gibco, California, US), 100 μg/mL streptomycin, and 100 U/mL penicillin (Meilunbio, Dalian, China) at 37°C in a 5% CO_2_ humidified incubator. MLg cells were treated with vehicle or recombinant IL-17A (ACMEC, Shanghai, China) for 12 h. For gene knockdown experiments, MLg cells were transfected with indicated negative control siRNA (NC-siRNA), *Il17ra*-siRNA (20 μM) (forward: 5′-GGCUGUGUGUCAAGUUCCATT-3′, reverse: 5′-UGGAACUUGACACACAGCCTT-3′) and *Cxcl1*-siRNA (20 μM) (forward: 5′- CCACUGCACCCAAACCGAAGUCAUA-3′, reverse: 5′- UAUGACUUCGGUUUGGGUGCAGUGG-3′) for 36 h, then treated with recombinant IL-17A (10ng/ml). All of siRNA were purchased from OBiO Technology (Shanghai). The efficacy of *Il17ra* and *Cxcl1* silencing were determined by quantitative reverse-transcriptase PCR (qRT-PCR). The following forward/reverse primer pairs were used: *Il17ra* enhancer (5′ to 3′): forward: CGGAGAATTAGTCCCTGTGTTG, reverse: GAACAGTCACTTCATACTCCTGG; *Cxcl1* enhancer (5′ to 3′): forward: ACTGCACCCAAACCGAAGTC, reverse: TGGGGACACCTTTTAGCATCTT. All of qRT-PCR primers were purchased from Sangon Biotech (Shanghai).

### Histopathological evaluation and immunofluorescence

The lung was fixed in 10% neutral buffered (pH 7.4) formalin for 24 h at room temperature. The lung tissues were embedded in paraffin, sliced into 5 μm, and stained with hematoxylin and eosin (H&E). For lung injury score, H&E slides were coded and scored from 0 (absent) to 4 (severe) for the following parameters: interstitial edema, hemorrhage, alveolar septal thickening, vascular congestion, and infiltration of the inflammatory cells by a pathologist in a blinded manner. The total “lung injury score’” was expressed as the sum of the scores for each parameter.

Multiplex immunofluorescence staining was performed using tyramide signal amplification (TSA) technology. Primary antibodies included two panels: the first panel was PDGFRA (AB134123, Abcam), CXCL1 (12335-1-AP, Proteintech), Ly6G (GB11229, Servicebio), and IL17RA (AB86487, Abcam); the second panel was PDGFRA (AB134123, Abcam), Cit-H3(AB281584, Abcam), MPO (22225-1-AP, Proteintech), and Ly6G (GB11229, Servicebio). Primary antibodies were sequentially applied, followed by horseradish peroxidase-conjugated secondary antibody incubation. Nuclei were stained with DAPI (G1012, Servicebio) after all the antigens above being labeled. The stained slides were scanned to obtain multispectral images using the Pannoramic MIDI imaging system (3D HISTECH).

All image analysis was performed using ImageJ Fiji. The positive signals in staining images were quantified from randomly selected sections of at least five fields for each sample using Image J 1.52 software (NIH, Bethesda, MD).

### Flow cytometry analysis

Lungs were minced into small pieces and mechanically dissociated by passing cells through 70 µM wire mesh, erythrocytes were removed by ACK lysis (Life Technologies, Carlsbad, CA), and 2x10^6^ cells were stained with fixable viability stain 700 (BD Biosciences) to exclude dead cells. For monocyte and neutrophil staining, samples were stained using anti-mouse antibodies against CD45-BUV395 7(30-F11), CD11b-BB515 (M1/70), CD11c-BV786(HL3), F4/80-BV421 (T45-2342), IA/IE-PE(M5/114.15.2), Ly6C-BV605 (AL-21), Ly6G-PerCP-Cy5.5(1A8), and SiglecF-A647 (E50-2440) for 30min at room temperature. All from BD Biosciences (Heidelberg, Germany). For transcription factor staining, cells were stained for surface markers, followed by fixation and permeabilization before nuclear factor staining using FOXP3 staining buffer set (eBioscience). For intracellular IL-17A staining, cells were incubated for 5 h in RPMI with 10% FBS, phorbol 12-myristate 13-acetate (PMA) (50 ng/mL; Sigma), ionomycin (500 ng/mL; Sigma) and GolgiStop (BD Biosciences), then samples were stained using anti-mouse antibodies against TCR-Vγ4-BUV737(GL3, BD Biosciences), CD127-BV421 (A7R34, BioLegend), CD3-BV510 (17A2, Biolegend), CD8a-BV650 (53-6.7,Biolegend), CD45-BUV395 (30-F11, BD Biosciences), NK1.1-PE-Cy7 (PK136, BD Biosciences), CD4-APC (RM4-5, BioLegend), RORγt-BV786 (Q31-378, BD Biosciences), IL-17A-PE (TC11-18H10, BD Biosciences), and Percp-Cy5.5 mouse lineage antibody cocktails containing: TER119 (BD Biosciences), CD11b (M1/70, BD Biosciences), CD11c (HL3, BD Biosciences) and Gr-1 (RB6-8C5, BioLegend). All multi-color flow cytometry data were acquired with a BD LSR Fortessa with Diva software and were analyzed by Flowjo version 10 (Tree Star).

### Neutrophil migration assays

Single-cell suspensions of bone marrow were obtained and neutrophils were isolated using the EasySep™ Mouse Neutrophil Enrichment Kit (STEMCELL Technologies). In brief, single-cell suspensions were incubated with biotin-antibody cocktail from isolation kits for 5min at 4°C, followed by incubation with anti-biotin microbeads for 10 min at 4°C. Cells purity was analyzed by flow cytometry analysis (at least 80%). For migration assays, neutrophils (5 × 10^5^) were placed in the upper chambers of transwell plate (5.0-mm pore diameter, Corning), bottom chambers were loaded with 600 μL of supernatants from MLg cultures. DMEM was used as a negative control, while DMEM with recombinant CXCL1 protein (GB300049, Servicebio) was used as a positive control. Plates were incubated for 2 h, the aspirates from the bottom chambers were mixed with 50 μL of precision count beads (BioLegend) to determine the relative density of neutrophil. The chemotactic index was calculated as fold of increase (or decrease) compared with medium or control groups.

### Single-cell RNA sequencing

Mice were euthanized at 48 h after PBS or induced DAD, then whole lungs were perfused with cold PBS. Lungs were aseptically removed and placed in 1 mL complete media (10% FBS, 1% Pen/Strep in RPMI). A single-cell suspension was prepared as noted above. CellRanger (v5.0) was used to aligned scRNA-Seq data to the mm10 mouse genome and quantify the gene expression count. Then, the output files were read into Seurat v4 (22) and cells with low quality were further excluded from the downstream analysis based on filtering by the following criteria (nFeature_RNA < 200 & nFeature_RNA > 7000 & percent.mt > 0.85). Dimension reductions include principal component analysis, clustering, and uniform manifold approximation and projection (UMAP) were then performed on the integration data. Immune cell subtype clustering and annotation were performed after sorted immune cells (CD45^+^, Ptprc) from the scRNA-seq data. Differentially expressed genes (DEGs) were identified for each cluster with FindAllMarkers function and cell types were assigned by using the CellMarker function. DEGs were used to perform enrichment analysis using gene set enrichment analysis (GSEA) on HALLMARK (mh.all.v2024.1.Mm.symbols.gmt), REACTOME (m2.cp.reactome.v2024.1.Mm.symbols.gmt), and GO-BP(m5.go.bp.v2024.1.Mm.symbols.gmt)gene sets in the GSEA software built locally. FDR was estimated by BH with a threshold of 0.05. Ligand-receptor communication using CellChat (23). Pseudobulk data were generated by pooling counts on library levels, these data were compared to obtained Pseudobulk DEGs. Ingenuity pathway analysis platform (IPA, Ingenuity Systems, QIAGEN) was used to predict the activity of pathways based on the observed gene expression changes. The NET formation score was evaluated using the AddModuleScore function in Seurat based on gene groups representing NET signature (24). This function determines the average module score based on the mean expression of the genes in the gene groups, by adjusting for the expression of other genes with comparable means throughout the dataset. Therefore, this score serves as an approximation of the NET formation in any given single cell.

### Statistical analysis

Data are analyzed using Prism v9.3.1 (GraphPad Software, La Jolla, CA). One-way analysis of variance followed by *post-hoc* Tukey’s multiple-comparison test or Kruskal–Wallis with Dunn’s multiple-comparison tests were used for multiple-group comparisons. Unpaired Student’s t-test or Mann–Whitney test was employed for comparisons between the two groups. Survival curves were generated using the Kaplan–Meier method, and differences among groups were compared using the Log-rank (Mantel–Cox) test to assess the significance of the survival differences. Correlations between parameters were calculated using the spearman correlation analysis and linear regression analysis as appropriate. *R* ^2^ indicates the regression coefficient. A *P* value of less than 0.05 was considered statistically significant, For all tests, ns: *P* ≥ 0.05, *P *<* 0.05, **P *<* 0.01, ***P *<* 0.001.

## Results

### Pathogenic neutrophil response in DAD immunopathology

**Figure 1:**
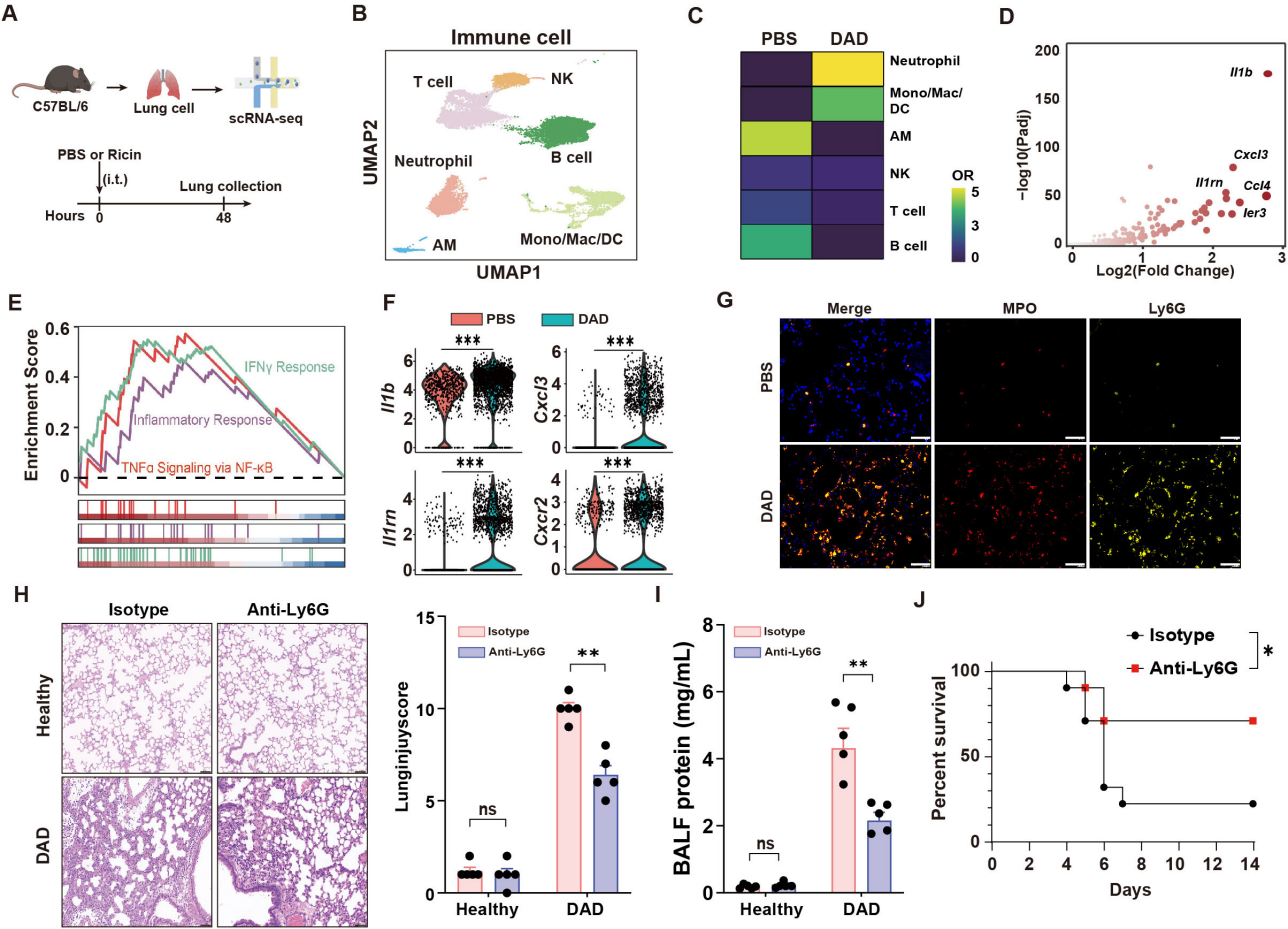
Neutrophil aggravates DAD immunopathology. **(A)** Schematic outline of scRNA-seq. **(B)** UMAP of immune cells showing annotated cell types. **(C)** Tissue prevalence of major cell types in the indicated group. **(D)** Volcano plot showing the upregulated DEGs in DAD neutrophil. **(E)** GSEA for the DEGs of neutrophils. **(F)** *Il1b*, *Il1rn*, *Cxcl3*, and *Cxcr2* expression levels in neutrophils. **(G)** Immunofluorescence MPO and Ly6G staining. Scale bar, 50 μm. **(H)** Representative H&E staining and quantification of lung injury score (n = 5). Scale bar, 50 μm. **(I)** Total protein levels in BALFs after treatment of Anti-Ly6G or isotype control (n = 5). **(J)** Kaplan–Meier analysis of survival in DAD mice after administration of Anti-Ly6G or isotype control (n = 10). Data are expressed as mean ± SEM. *P *<* 0.05, **P *<* 0.01, ***P *<* 0.001.

Using scRNA-seq, we characterized the cellular landscape of lung tissues in a murine model of DAD induced by intratracheal administration of aerosolized ricin (**Figure 1A**). Through the application of established gene markers and cell type-specific Gene Ontology (GO) enrichment analysis, we identified six distinct immune cell populations in both PBS-treated and ricin-induced DAD lungs (hereafter referred to as PBS and DAD lungs, respectively) (**Figure 1B**, **Supplementary Figures S1A–C**). Notably, a significant expansion of neutrophil was observed in DAD lungs compared to PBS lungs (**Figure 1C**), underscoring their potential role in the immunopathology of DAD.

We compared the transcriptional profiles of neutrophil from DAD lungs and PBS lungs, revealing markedly upregulated inflammatory genes in neutrophil from DAD lungs (**Figure 1D**). GSEA further demonstrated robust activation of proinflammatory pathways in neutrophil from DAD lungs, including “IFNγ response” and “TNFα signaling via NF-κB” (**Figure 1E**). We observed upregulated expression of inflammatory mediators (*Il1b*, *Cxcl3*) and their cognate receptors (*Il1rn*, *Cxcr2*) in DAD-derived neutrophils (**Figure 1F**), suggesting a self-amplifying signaling cascade that perpetuates neutrophil recruitment and activation. Additionally, immunofluorescent staining corroborated these findings, showing elevated densities of Ly6G (a neutrophil marker) and MPO (a marker of neutrophil activation) in DAD lungs compared to PBS lungs (**Figure 1G**), indicative of a hyperactivated neutrophil state.

To investigate the functional role of neutrophils in DAD lungs, we employed a Ly6G-specific monoclonal antibody (Anti-Ly6G) for neutrophil depletion (**Supplementary Figure S2A**). The depletion of neutrophils was effective at 48 h after DAD induction as demonstrated by flow cytometry (**Supplementary Figure S2B**, the gating strategy is illustrated in **Supplementary Figure S2C**). Histopathological analysis of DAD lungs showed Anti-Ly6G treatment attenuated lung injury (**Figure 1H**). The protein levels in BALFs, an indicator of lung damage, were lower in neutrophil depletion mice (**Figure 1I**). Moreover, mice with neutrophil depletion demonstrated higher survival rates (**Figure 1J**). Collectively, this work elucidates the contributions of recruited neutrophils to lung immunopathology during DAD progression.

### NETs drive tissue damage in DAD lungs

**Figure 2:**
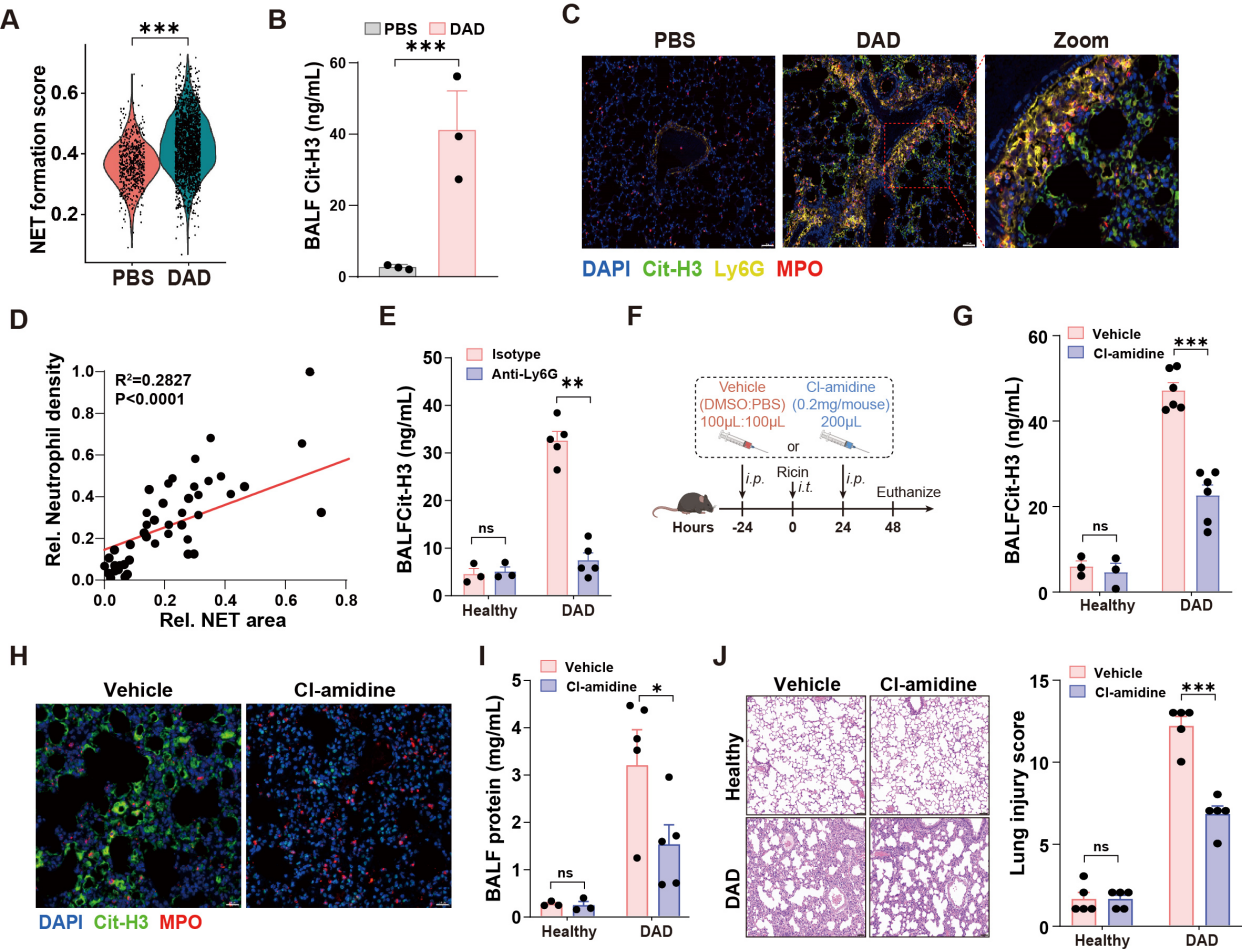
NET mediates immunopathological lung injury. **(A)** The NET formation score in neutrophil of scRNA-seq data. **(B)** Quantification of Cit-H3 levels in BALFs from PBS and DAD lungs (n = 3). **(C)** Representative immunofluorescence staining of Cit-H3 (green) and Ly6G (yellow) in DAD lungs. Scale bar, 50 μm. **(D)** Correlation of neutrophil density and NET areas in DAD lungs. **(E)** Quantification of Cit-H3 levels in BALFs from DAD mice treated with Anti-Ly6G or isotype control (n = 3-5). **(F)** Experimental design for **(G-J)**. **(G)** Quantification of Cit-H3 levels in BALFs (n = 3-6). **(H)** Representative immunofluorescence staining of Cit-H3 (green) and MPO (red). Scale bar, 20 μm. **(I)** Quantification of total protein levels in BALFs in BALFs (n = 3-6). **(J)** Representative H&E staining and quantification of lung injury score (n = 5). Scale bar, 50 μm. Data are expressed as mean ± SEM. ns, not significant. *P *<* 0.05, **P *<* 0.01, ***P *<* 0.001.

Next, we performed a comprehensive functional analysis of neutrophils in DAD lungs. First, the NET score was significantly elevated in neutrophil from DAD lungs (**Figure 2A**). Second, levels of Cit-H3, a well-established NET marker, were markedly increased in BALFs from DAD lungs (**Figure 2B**). Third, immunofluorescent staining for Cit-H3 revealed enhanced NET formation in DAD lungs relative to PBS controls (**Figure 2C**). Notably, we identified a significant correlation between neutrophil density and NET areas in DAD lungs (**Figure 2D**), while the Cit-H3 levels in BALFs and in lung tissue were significantly reduced after neutrophil depletion (**Figure 2E**, **Supplementary Figure S2D**), confirming that extracellular traps predominantly originate from neutrophil.

To investigate the role of NETs in DAD immunopathogenesis, we administered Cl-amidine, a PAD4 inhibitor that blocks Cit-H3 production, to DAD mice (**Figure 2F**). Cl-amidine treatment significantly reduced Cit-H3 levels in BALFs and diminished NET formation in lungs (**Figures 2G, H**). Additionally, Cl-amidine administration led to decreased BALF protein levels and attenuated lung injury scores (**Figures 2I, J**). Further, we directly degraded NETs using DNase I, which resulted in reduced NET presence in DAD lungs (**Supplementary Figures S3A, B**). Notably, DNase I treatment decreased BALF total protein levels, even though Cit-H3 levels in BALFs remained unchanged (**Supplementary Figures S3C, D**). Together, these findings highlight the critical contribution of neutrophil-derived NETs to immunopathological injury in DAD lungs.

### IL-17A signaling orchestrates neutrophil recruitment and NET formation

**Figure 3:**
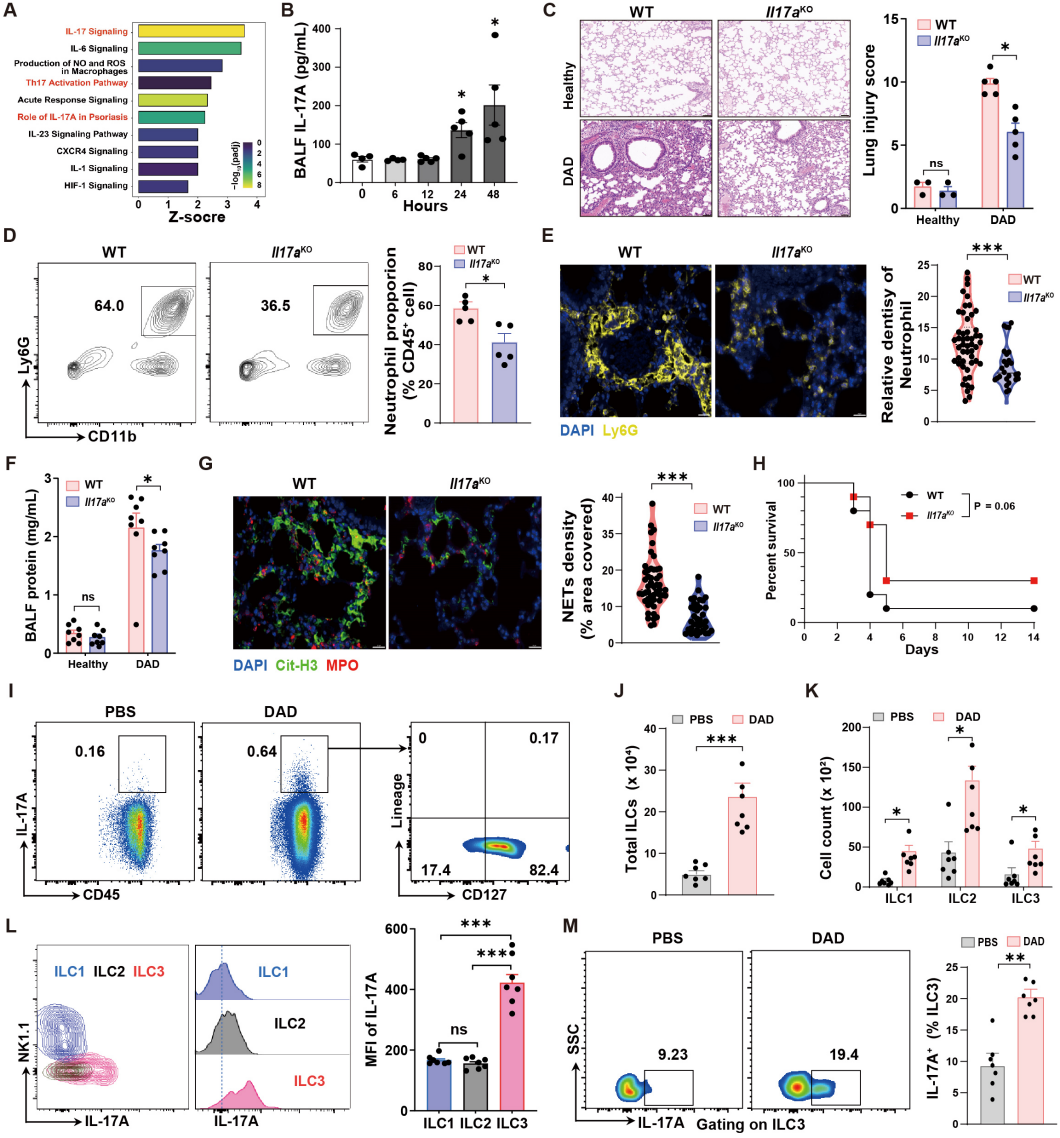
IL-17A deteriorates lung damage by promoting neutrophil recruitment and NET formation. **(A)** IPA pathway analysis of scRNA-seq data. **(B)** Quantification of BALF IL-17A levels during DAD progression (n = 4-5). **(C)** Representative H&E staining and quantification of lung injury score (n = 3-5). **(D)** Representative FCM plots for neutrophil in BALFs and quantification (n = 5). **(E)** Representative immunofluorescence staining for Ly6G (yellow) and quantification per field of view (n = 50). Scale bar, 50 μm. **(F)** Quantification of total protein levels in BALFs (n = 8). **(G)** Representative immunofluorescence staining for Cit-H3 (yellow) and quantification of NET density per field of view (n = 50). Scale bar, 20 μm. **(H)** Kaplan–Meier analysis of survival (n = 10). **(I)** Representative FCM plots, percentages of CD45^+^ IL-17A^+^ cells in PBS-operated and DAD mice, and percentages of lung IL-17A^+^ ILCs (IL-17A^+^ CD127^+^ Lineage^-^). **(J)** Quantification of total cell number of ILCs in PBS-operated and DAD mice (n = 7). **(K)** Quantification of cell number of ILC subsets in PBS-operated and DAD mice (n = 7). **(L)** Quantification of lung IL-17A^+^ cells in ILC subsets (n = 7). **(M)** Representative FCM plots and percentages of IL-17A^+^ ILC3s in the lungs of PBS-operated and DAD mice (n = 7). Data are expressed as mean ± SEM. ns, not significant. *P *<* 0.05, **P *<* 0.01, ***P *<* 0.001.

To investigate the signaling pathways driving neutrophil recruitment and NET formation, we first performed IPA analysis to predict the active pathways. This analysis identified IL-17 signaling as a prominent pathway enriched in DAD lungs (**Figure 3A**). Consistent with this finding, a significant increase in IL-17A levels was observed in BALFs within 48 h during DAD progression (**Figure 3B**).

Next, we investigated potential function of IL-17A in DAD lungs with *Il17a* ^KO^ mice. Compared to WT mice, *Il17a* ^KO^ mice demonstrated decreased pathological scores, indicative of less lung tissue damage (**Figure 3C**). This corresponded with reduced neutrophil accumulation in both BALFs and lung tissues relative to WT mice (**Figures 3D, E**). Additionally, the total protein levels in BALF and NET formation in lung were significantly reduced in *Il17a* ^KO^ mice (**Figures 3F, G**). Furthermore, *Il17a* ^KO^ mice exhibited a trend toward improved survival following DAD induction, though this difference did not reach statistical significance (P = 0.06) compared to WT controls (**Figure 3H**). These findings indicate that IL-17A serves as a key driver of neutrophilic inflammation, tissue injury, and NET formation in DAD pathogenesis. To assess the therapeutic potential of targeting IL-17A, we administered a neutralizing antibody against IL-17A (**Supplementary Figure S4A**). IL-17A neutralization also led to reduced neutrophil infiltration and alleviated BALF protein, indicating the reduced inflammation and lung injury, respectively (**Supplementary Figures S4B, C**).

To investigate the cellular sources of IL-17A production in DAD lungs, we analyzed several cell types that have been confirmed as sources of IL-17A, including γδ T cells, natural killer cells, natural killer T cells, CD4^+^ T cells, CD8^+^ T cells, and ILCs, using FCM analysis (**Supplementary Figure S5**). Notably, we found that ILCs stood out as the dominant cellular source of IL-17A production in DAD lungs (**Figure 3I**). We also demonstrated a substantial increase in the total ILC population (**Figure 3J**) and elevated numbers of ILC1, ILC2, and ILC3 subsets (**Figure 3K**). Among these subsets, ILC3 constituted the dominant cellular source of IL-17A that induced in DAD lungs (**Figures 3L, M**). Collectively, these findings demonstrate the ILC3-derived IL-17A as a central regulator in driving neutrophil recruitment and NET formation, thereby exacerbating immunopathological injury in DAD lungs.

### IL-17A-neutrophil-NET feedback loop sustains inflammatory responses

**Figure 4:**
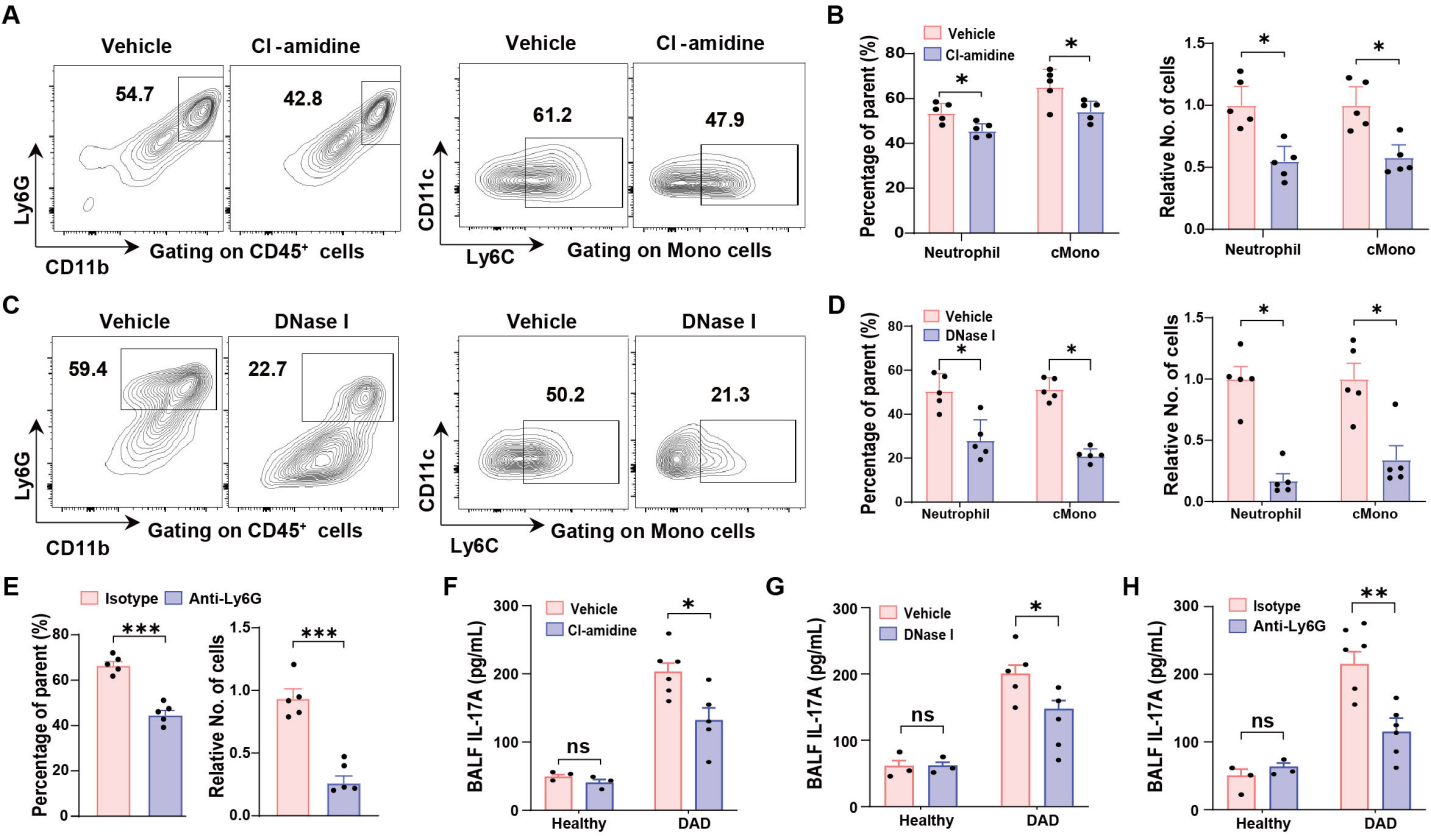
IL-17A-neutrophil-NET positive feedback loop. **(A, B)** Representative FCM plots for neutrophil (left) and classical monocyte (cMono, right) identification in the lungs from DAD mice treated with vehicle or Cl-amidine. **(B)** Quantification of proportions (left) and relative cell numbers (right) of neutrophil and cMono, related to **(A)** (n = 5). **(C)** Representative FCM plots for neutrophil (left) and classical monocyte (cMono, right) identification in the lungs from DAD mice treated with vehicle or DNase I. **(D)** Quantification of proportions (left) and relative cell numbers (right) of neutrophil and cMono, related to **(C)** (n = 5). **(E)** Quantification of proportions (left) and relative cell numbers (right) of cMono in the lungs from DAD mice treated with isotype or Anti-Ly6G (n = 5). **(F)** Quantification of IL-17A levels in BALFs from healthy and DAD mice treated with vehicle or Cl-amidine (n = 3-5). **(G)** Quantification of IL-17A levels in BALFs from healthy and DAD mice treated with vehicle or DNase I (n = 3-5). **(H)** Quantification of IL-17A levels in BALFs from healthy and DAD mice treated with isotype or Anti-Ly6G (n = 3-5). Data are expressed as mean ± SEM. ns, not significant. *P *<* 0.05, **P *<* 0.01, ***P *<* 0.001.

First, we observed that targeting NETs with either Cl-amidine or DNase I significantly reduced both the proportions and absolute counts of neutrophils and classical inflammatory monocytes (cMono) in DAD lungs (**Figures 4A–D**, gating strategy is shown in **Supplementary Figure S2C**). Similarly, neutrophil depletion via Anti-Ly6G treatment led to a decrease in cMono populations (**Figure 4E**). Second, interventions targeting NETs or neutrophils—including Cl-amidine, DNase I, and anti-Ly6G—resulted in reduced IL-17A levels in BALFs (**Figures 4F–H**), suggesting that neutrophil accumulation and NET formation enhance IL-17A production in a self-reinforcing manner. Collectively, these findings demonstrate that NET formation plays a critical role in amplifying inflammatory responses by promoting neutrophil recruitment and sustaining an IL-17A-neutrophil-NET feedback loop during DAD progression.

### Fibroblast-derived CXCL1 mediates IL-17A-dependent neutrophil recruitment

**Figure 5:**
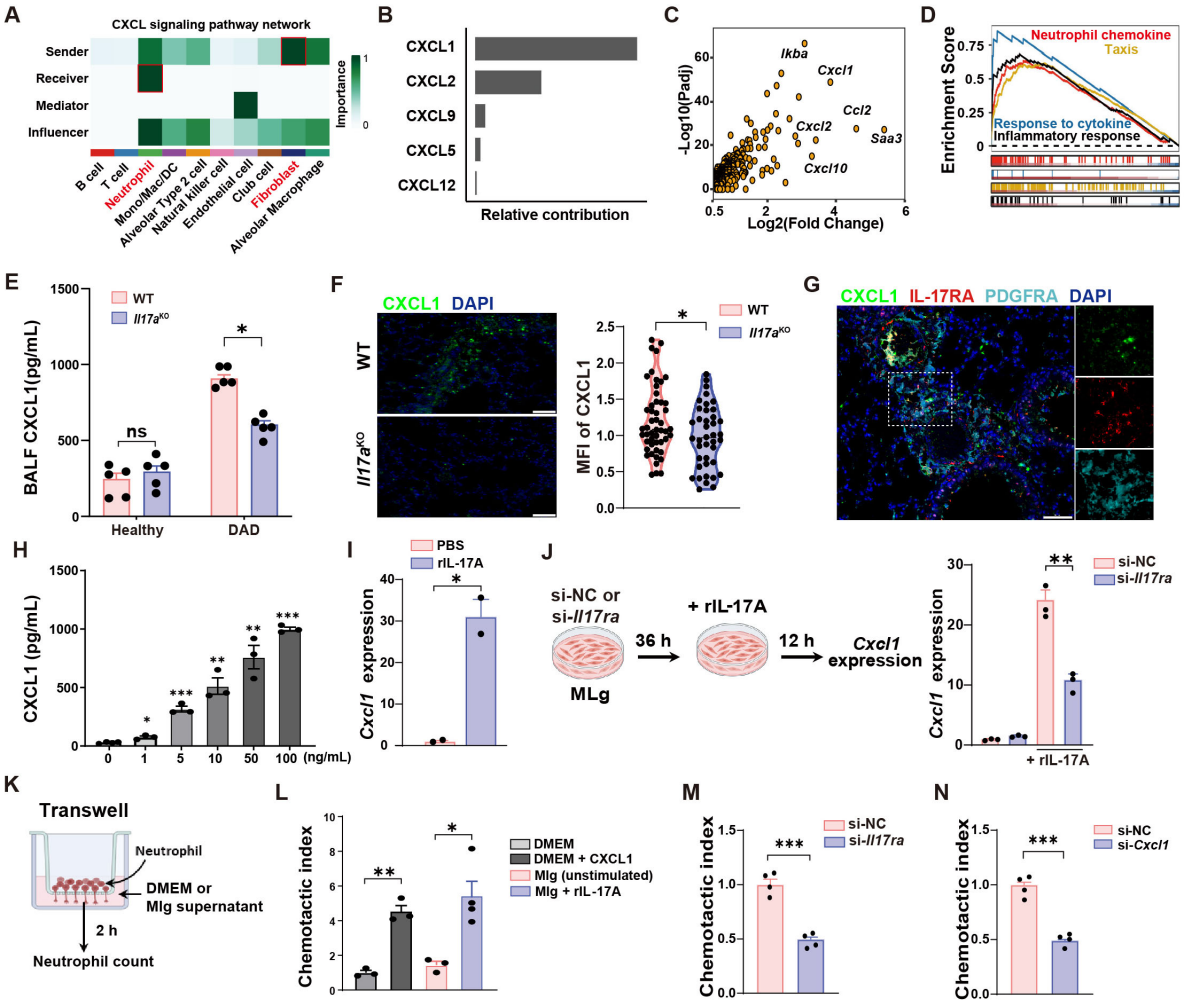
IL-17A promotes neutrophil recruitment by increasing CXCL1 secretion from fibroblasts. **(A)** Intercellular crosstalk analysis between cell types of DAD datasets in scRNA-seq data. **(B)** Top five candidates enriched in the CXCL signaling pathway. **(C)** Volcano plot showing the upregulated DEGs in DAD fibroblast. **(D)** GSEA for the DEGs of fibroblast. **(E)** Quantification of CXCL1 protein levels in BALFs from WT and *Il17a* ^KO^ mice (n = 5). **(F)** Representative immunofluorescence staining for CXCL1 (green) and quantification per field of view (n = 50). Scale bar, 50 μm. **(G)** Co-staining of PDGFRA, IL-17RA, and CXCL1 in DAD lungs. Scale bar, 50 μm. **(H)** MLg cells were treated with IL-17A (0, 1, 5, 10, 50, 100 ng/mL) for 12 h and the protein levels of CXCL1 in Mlg supernatants were examined by ELISA (n = 3 - 4). **(I)** MLg cells were treated with 10 ng/mL IL-17A for 12 h and the gene expressions of CXCL1 were examined by qRT-PCR (n = 2). **(J)** Experimental design (left) and quantification (right) of *Cxcl1* expression of si-NC or si-*Il17ra*- treated MLg cells stimulated with IL-17A (n = 3). **(K)** Experimental design for neutrophil transwell assays. **(L)** Corresponding statistical analysis of MLg chemotactic abilities after IL-17A stimulation (n = 3-4). DMEM with or without CXCL1 (100 ng/mL) were used as a positive and negative control. **(M)** MLg chemotactic abilities after IL-17A-stimulated MLg cells under *Il17ra*-siRNA blockage (n = 4). **(N)** MLg chemotactic abilities after IL-17A-stimulated MLg cells under *Cxcl1*-siRNA blockage (n = 4). Data are expressed as mean ± SEM. ns, not significant. *P *<* 0.05, **P *<* 0.01, ***P *<* 0.001.

To explore the underlying mechanism behind the increased neutrophil infiltration in DAD lungs, we performed an in-depth analysis of the intercellular crosstalk using scRNA-seq data, and identified robust fibroblast-neutrophil interactions mediated by the CXCL chemokine network in DAD lungs (**Figure 5A**), with CXCL1 exhibiting the highest interaction scores (**Figure 5B**). Consistent with this, fibroblasts in DAD lungs exhibited widespread changes in gene expression, including upregulation of several chemokine genes, such as *Cxcl1* (**Figure 5C**). Furthermore, GSEA revealed significant enrichment of pathways associated with inflammatory responses, neutrophil chemotaxis, and cytokine signaling in DAD fibroblasts (**Figure 5D**). These findings indicate that fibroblasts likely play an active role in promoting neutrophil recruitment through CXCL1 production during DAD progression.

To further validate this hypothesis, we conducted *in vivo* experiments. We observed that *Il17a* ^KO^ mice exhibited significantly reduced CXCL1 levels in DAD lungs compared to WT controls (**Figure 5E**). Immunofluorescent staining further corroborated these findings, demonstrating diminished CXCL1 fluorescence intensity in *Il17a* ^KO^ DAD lung (**Figure 5F**). Additionally, we confirmed the presence of PDGFRA^+^ fibroblasts in DAD lungs by co-staining of CXCL1 and IL-17RA proteins (**Figure 5G**), suggesting that IL-17A might regulate the CXCL1 expression in fibroblasts.

To understand whether IL-17A affects the expression of CXCL1 in fibroblasts, we performed *in vitro* experiments with a mouse lung fibroblast cell line (MLg). We found that CXCL1 protein levels in MLg supernatants were increased upon recombinant IL-17A stimulation in a dose-dependent manner (**Figure 5H**). To avoids potential non-specific or cytotoxic effects of high concentration, we choose 10 ng/mL IL-17A for subsequent experiments. At this concentration, IL-17A significantly upregulated gene expression of CXCL1, detected by qRT-PCR in MLg cells (**Figure 5I**). Furthermore, treatment of *Il17ra*-siRNA significantly suppressed the gene expression and secretion of CXCL1 in Mlg cell after IL-17A stimulation (**Figure 5J**, **Supplementary Figures S6A, B**). These data suggested that CXCL1 production in MLg cells is mechanistically linked to IL-17A signaling through the IL-17RA receptor. To ascertain whether MLg-derived CXCL1 after IL-17A stimulation modulates chemotaxis of neutrophils, we performed neutrophil transwell assays (**Figure 5K**). We found that MLg cells stimulated with IL-17A were highly effective in enhancing neutrophil migration (**Figure 5L**). However, neutrophil migration was inhibited by *Il17ra*- or *Cxcl1*-siRNA treatment (**Figures 5M, N**, **Supplementary Figures S6C, D**). These findings demonstrated that IL-17A induced CXCL1 production from fibroblasts was responsible for neutrophil recruitment.

## Discussion

DAD, a severe and fatal manifestation of acute lung injury, poses a significant public health challenge. Building on our previous work characterizing crosstalk between fibroblast and neutrophil in DAD progression (18), we investigated the cellular and molecular mechanisms of neutrophils in driving immunopathology in this study.” the cellular and molecular mechanisms of neutrophils in driving immunopathology. We found that neutrophil in DAD lungs adopted activated phenotype and enhanced NET formation, which further exacerbated inflammation and tissue injury. while IL-17A signaling was identified as a key driver of neutrophil recruitment and NET formation. Importantly, IL-17A stimulated lung-resident fibroblasts to produce CXCL1, a potent neutrophil chemoattractant, establishing a feedforward loop that perpetuated neutrophilic inflammation. Furthermore, ILC3 served as the dominant cellular source of IL-17A in DAD lungs. This study delineates a pathogenic IL-17A-neutrophil-NET axis that amplifies lung immunopathology in DAD, providing mechanistic insights into the interplay between immune and structural cells in acute lung injury.

Neutrophil represented the first waves of immune cells that migration to lung tissues after encountering insults (25). NET formation is a unique form of programmed apoptosis by neutrophils, where decondensed chromatin and antimicrobial granules are released into the extracellular space in a PAD4-dependent manner (3, 26–28). The reduction in NET formation and associated tissue damage following PAD4 inhibition (Cl-amidine) or NET degradation (DNase I) further supports the notion that NETs are not merely bystanders but active drivers of inflammation and injury. These findings are in line with growing evidence implicating NETs in the pathogenesis of inflammatory diseases (29, 30). However, the precise molecular mechanisms that how NETs exacerbate DAD progression, particularly their interactions with other immune and structural cells, warrant further investigation.

Fibroblast-immune cell interactions have drawn increasing attention in immunological research. Recent studies have identified diverse fibroblast subsets in healthy and injured tissues, demonstrating the multifaceted roles of resident fibroblasts in maintaining tissues homeostasis and reshaping the local immune landscape (9, 31). Thus, targeting lung fibroblasts might be an efficient strategy to coordinate an immune microenvironment than targeting a single population of immune cells for treating inflammatory lung disease. Although numerous studies have implicated a role for IL-17A signaling in immune cell behavior, the potential for inflammatory signals to drive fibroblast activation is less explored. In support of this hypothesis, we showed that fibroblast produced chemokine CXCL1 upon IL-17A stimulation, and mediated neutrophil recruitment, thus promoting an immune-fibroblasts niche formation. It is worth noting that genetic IL-17A depletion, or its systemic inhibition confer an “all-or-none” effect, that might lead potential confounding factors. Thus, future study that generated mice that lack IL-17A receptor in fibroblast, are needed to explore the specifical role of IL-17A in fibroblasts.

At last, our current study showed that lung ILC3 represents the crucial cellular source of IL-17A, which is consisted with previous studies (32, 33). ILC3 plays clear roles in regulating mucosal immunity and tissue homeostasis. However, the involvement of ILC3 in DAD lungs is less well-defined. IL-17A from ILC3 is recognized as a key driver of neutrophilic inflammation that play pathogenic role in lung disease (34). This raises the intriguing possibility that manipulating ILC3 represent promising new targets for treating lung injury disease.

In summary, this study delineates a pathogenic IL-17A-neutrophil-NET axis that amplifies lung immunopathology after ricin-induced DAD. Neutrophils, via NET formation, and fibroblasts, via CXCL1 production, act as both effectors and regulators of IL-17A-driven inflammation, while ILC3s serve as a key cellular source of IL-17A. By identifying IL-17A, NETs, and fibroblast-derived CXCL1 as key contributors to disease progression, our findings offer potential therapeutic targets for mitigating lung injury in DAD and related neutrophilic inflammatory disorders.

## Data Availability

The data presented in the study are deposited in the China National Center for Bioinformation repository, accession number PRJCA036269.
