# Supplementary material for: IL-17A drives a fibroblast-neutrophil-NET axis to exacerbate immunopathology in the lung with diffuse alveolar damage
Source: Front Immunol. 2025 Jun 11;16:1574246. doi: 10.3389/fimmu.2025.1574246 (PMC12187750; doi:10.3389/fimmu.2025.1574246)
Supplement: Supplementary file 1 [file Table1.docx]

# Supplemental Figures


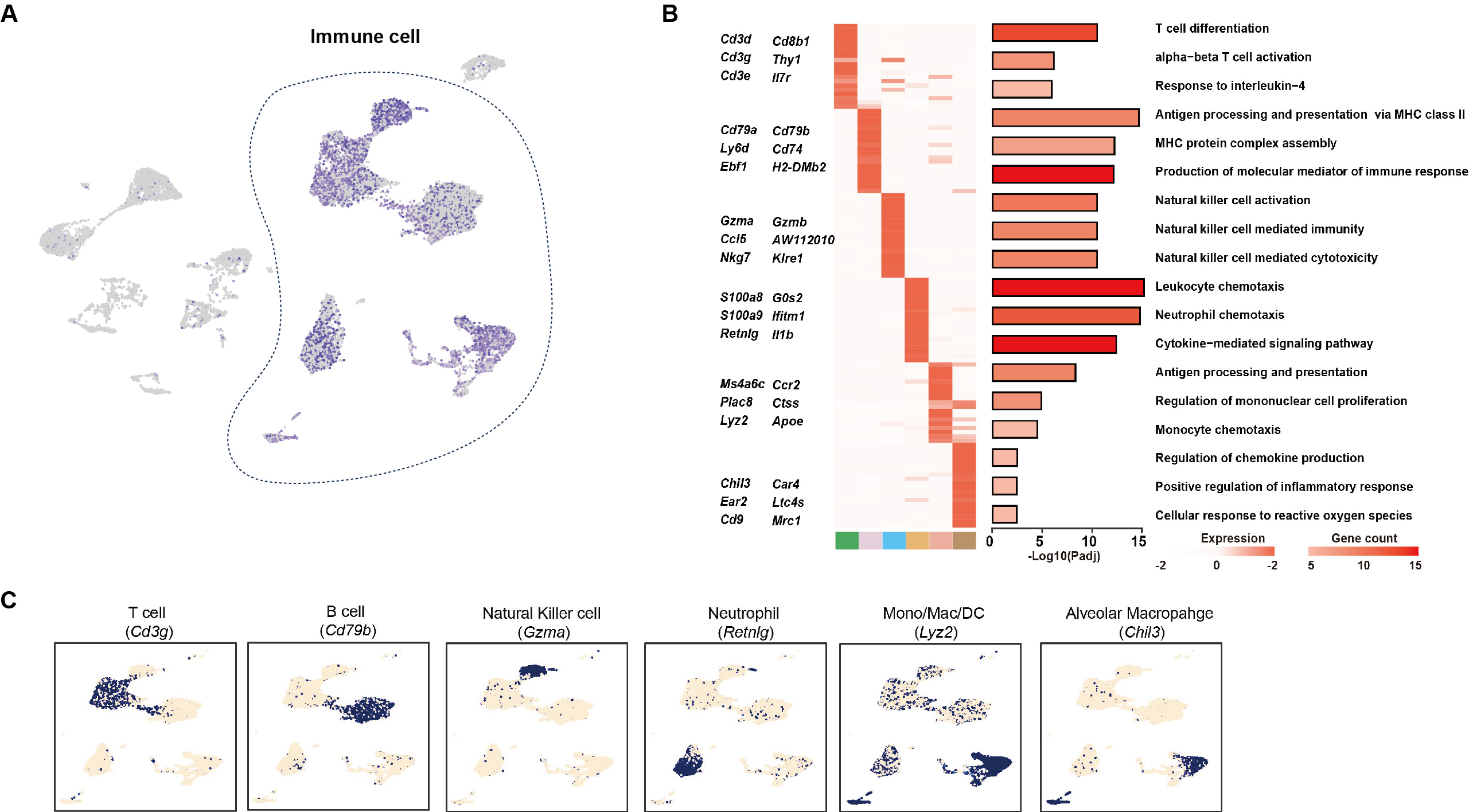


Supplemental Figure 1. scRNAseq of the major lung cell types. (A) UMAP of the entire scRNA-seq dataset showing CD45^+^ (encoding gene *Ptprc*) immune cell types. (B) Top DEGs and enriched biological processes for distinct cell types in immune cell types. (C) Cell markers for identifying major immune cell types.


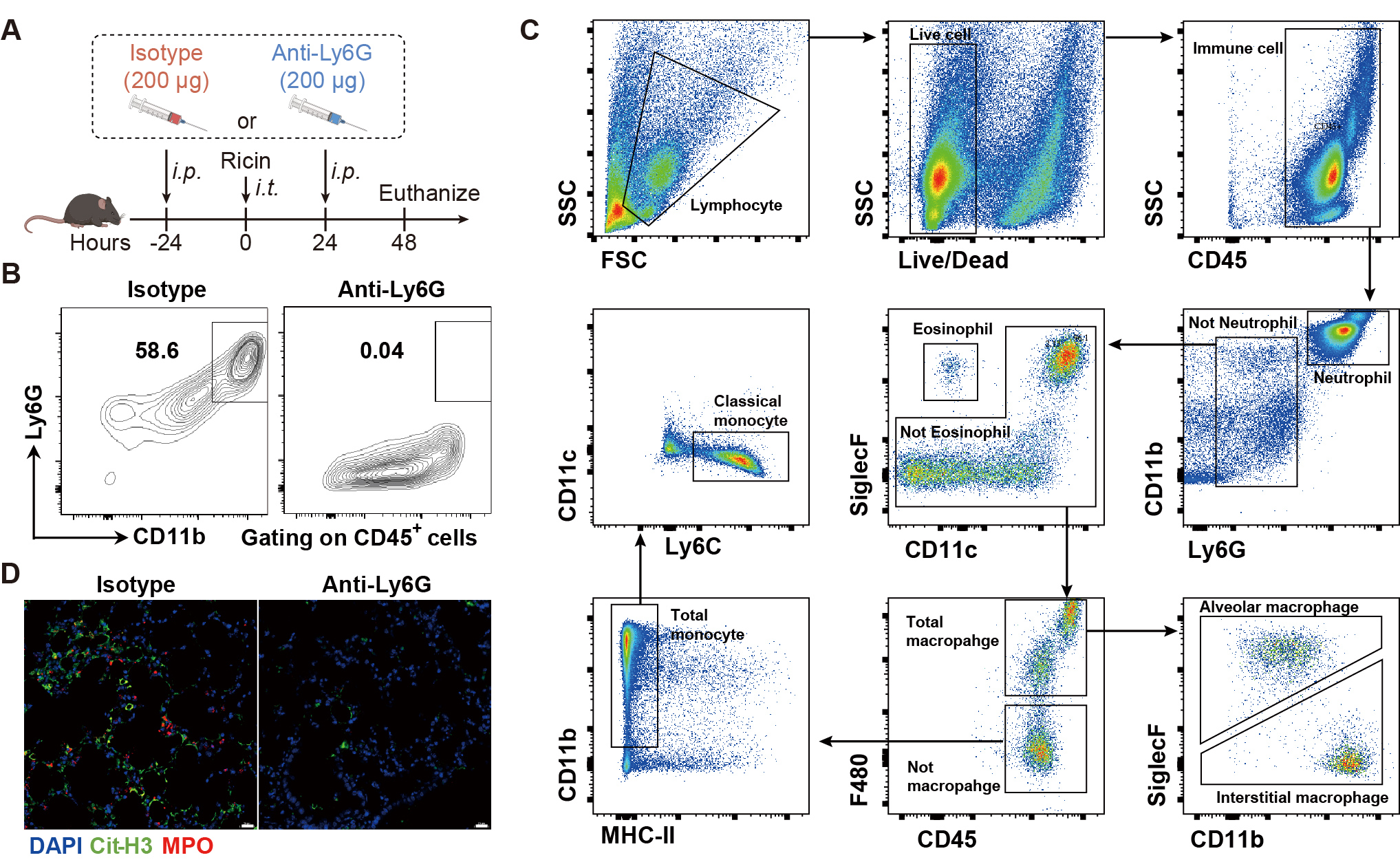


Supplemental Figure 2. NET formation in neutrophils. (A) Experimental design for (B-D). (B) Representative flow plots for neutrophil in isotype and Anti-Ly6G treatment group. (C) The FCM gating strategy for identifying major myeloid cell types. (D) Representative immunofluorescence staining of Cit-H3 (green) and MPO (red) in DAD lungs, scale bar 50 μm.


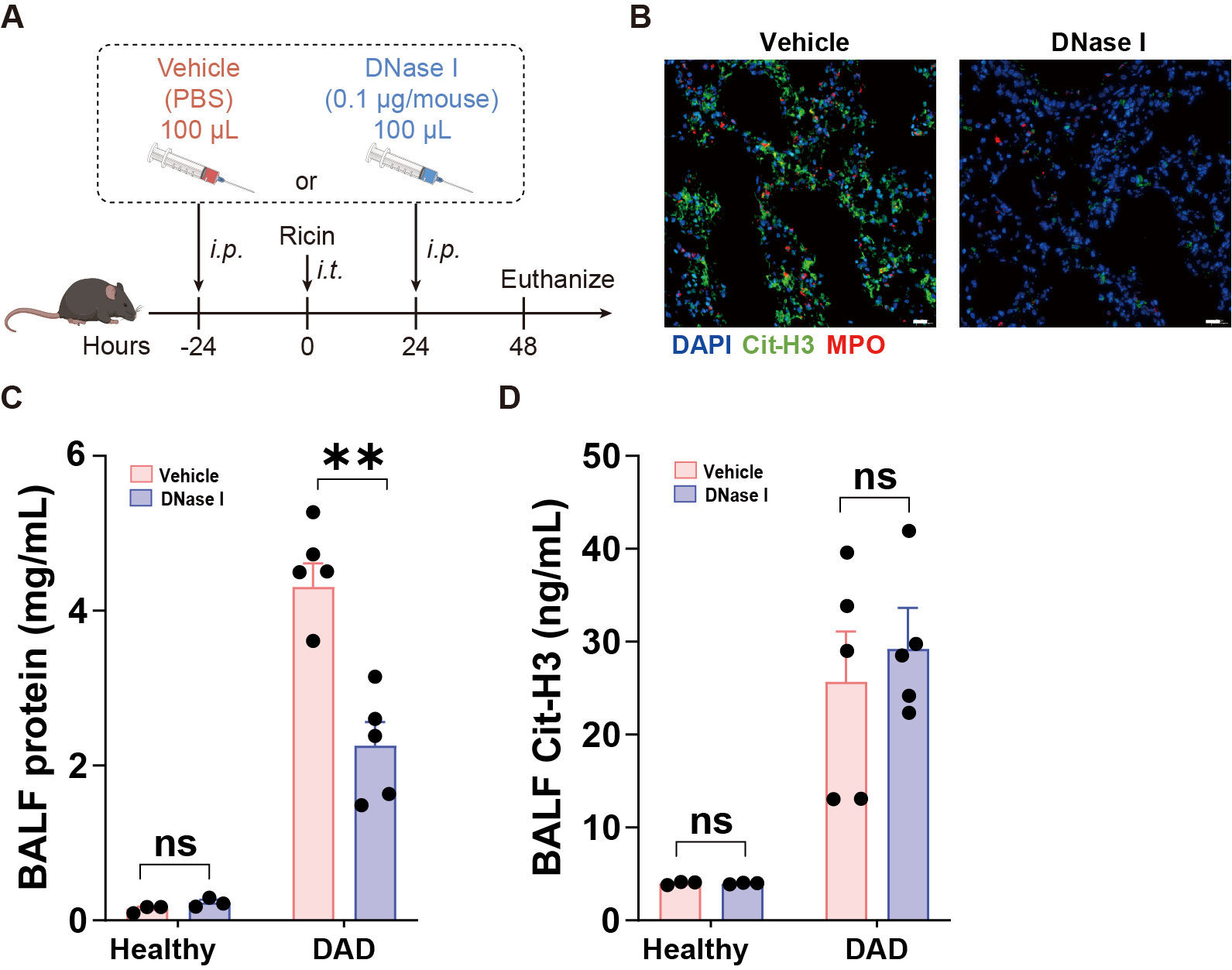


Supplemental Figure 3. DNase I treatment attenuated NET-mediated lung injury. (A) Experimental design for (B-D). (B) Representative immunofluorescence staining of Cit-H3 (green) and MPO (red), scale bar 50 μm. (C) Quantification of total protein levels in BALFs (n = 3 - 5). (D) Quantification of Cit-H3 levels in BALFs (n = 3 - 5). Data are expressed as mean ± SEM. ns, not significant. **P *<* 0.01.


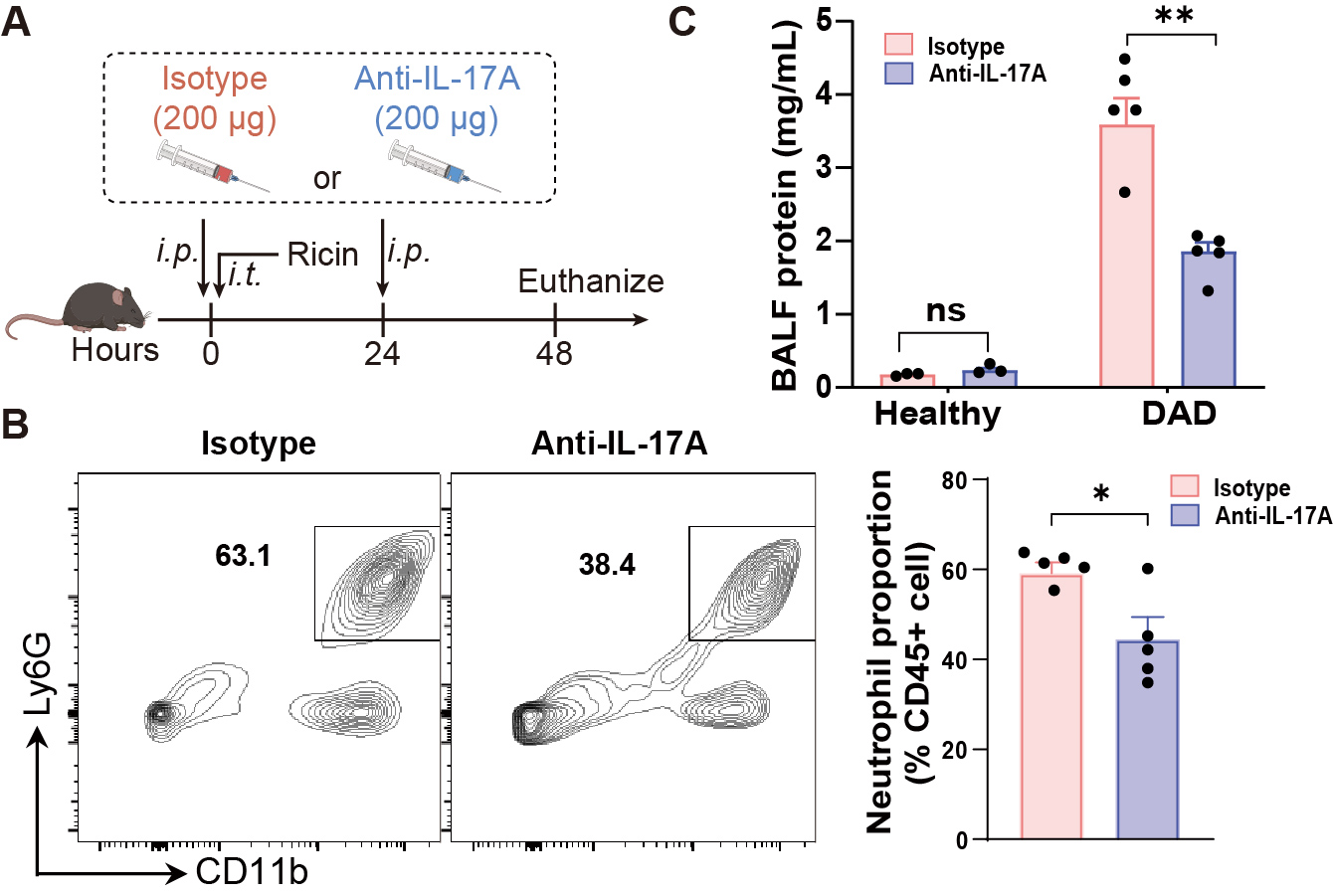


Supplemental Figure 4. IL-17A neutralizing attenuated neutrophil accumulation and lung damage. (A) Experimental design for (B-C). (B) Representative FCM plots for identification of neutrophil and quantification of neutrophil proportion (n = 5). (C) Quantification of total protein levels in BALFs (n = 3 - 5). Data are expressed as mean ± SEM. ns, not significant. *P *<* 0.05, **P *<* 0.01.


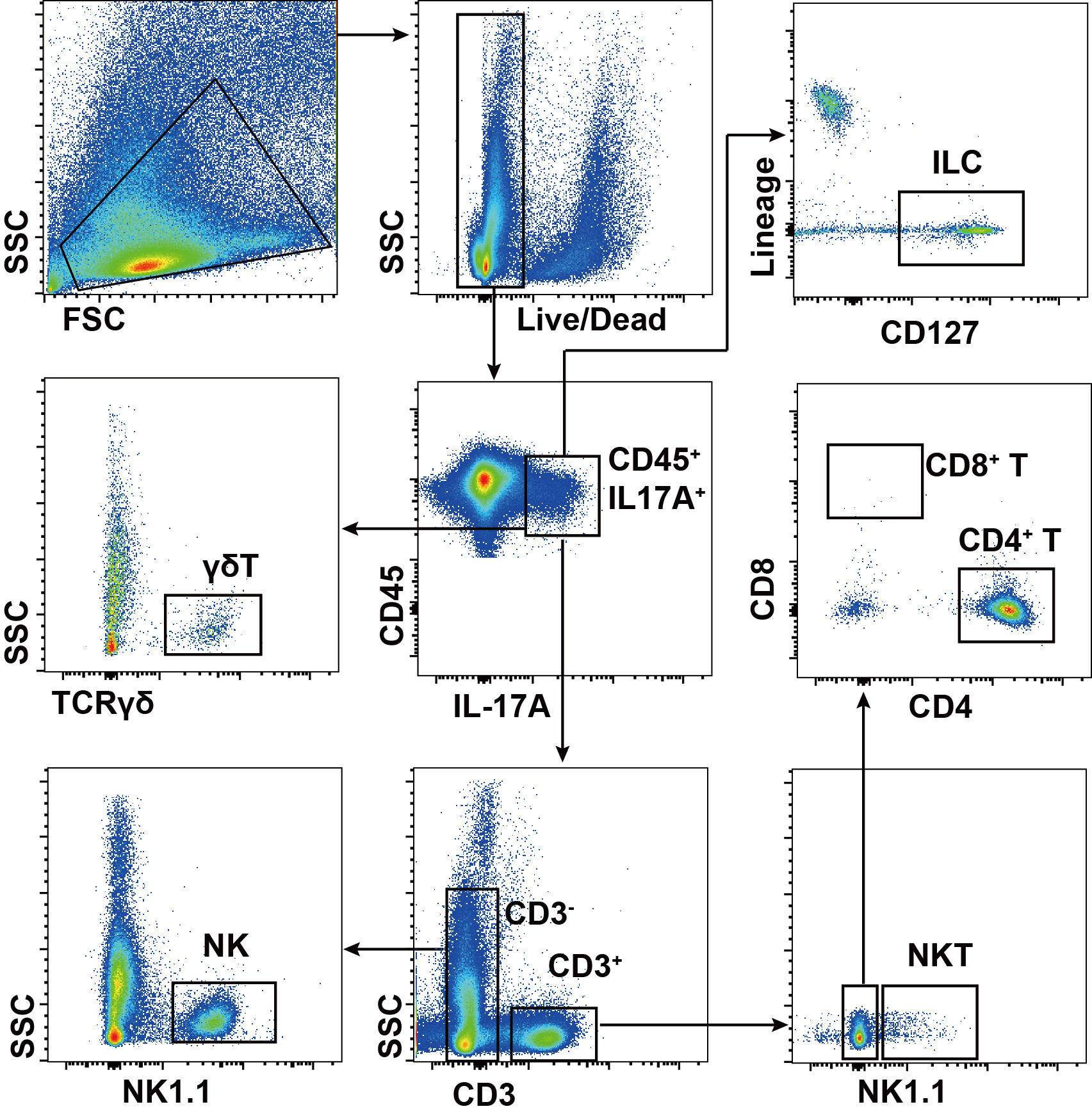


Supplemental Figure 5. The FCM gating strategy for identifying major cellular source of IL-17A production.


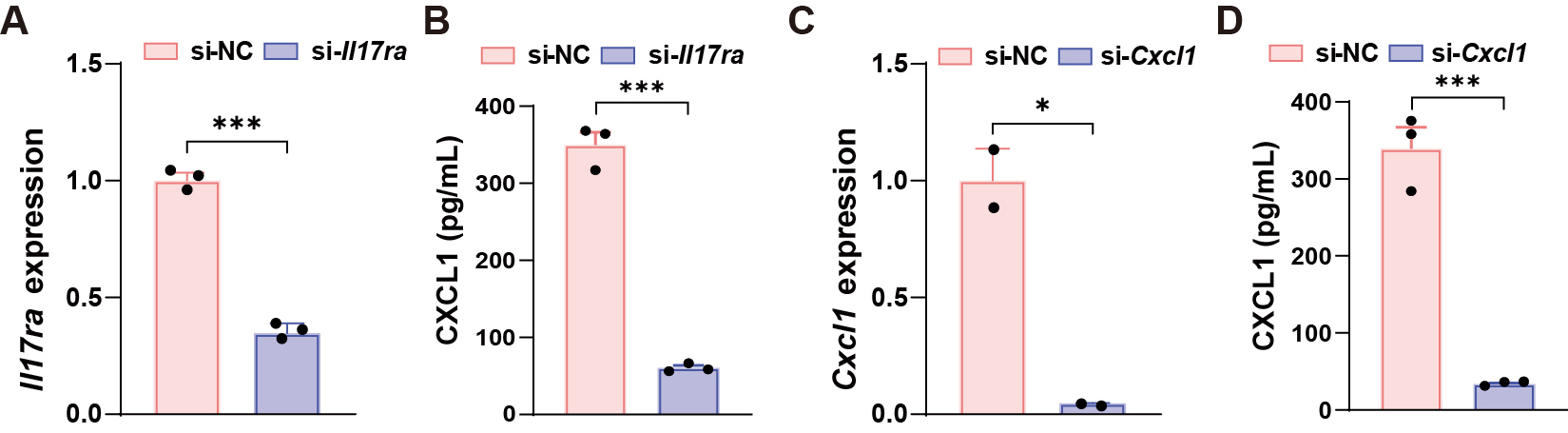


Supplemental Figure 6. Silencing efficacy of siRNA was determined by qRT-PCR and ELISA. (A) Gene expression levels of *Il17ra* after *Il17ra* siRNA silencing in MLg cells (n = 3). (B) CXCL1 protein levels after *Il17ra* siRNA silencing in MLg supernatants after IL-17A stimulation (n = 3). (C) Gene expression levels of *Cxcl1* after *Cxcl1* siRNA silencing in MLg cells (n = 2). (D) CXCL1 protein levels after *Cxcl1* siRNA silencing in MLg supernatants after IL-17A stimulation (n = 3).
